# Context-dependent thermolability of sex determination in a lacertid lizard with heteromorphic sex chromosomes

**DOI:** 10.1242/bio.059967

**Published:** 2023-05-16

**Authors:** Alexander Hansson, Erik Wapstra, Geoffrey M. While, Willow R. Lindsay, Mats Olsson

**Affiliations:** ^1^ Department of Biological and Environmental Sciences, University of Gothenburg, Box 463, 405 30, Gothenburg, Sweden; ^2^ School of Natural Sciences, University of Tasmania, Private Bag 55, Hobart, TAS, 7001 Australia

**Keywords:** Sex determination, Development, Incubation temperature, Reptile

## Abstract

Developmental conditions can profoundly impact key life history traits of the individual. In cases where offspring sex is driven by developmental reaction norms, permanent changes to the phenotype can fundamentally alter life history trajectories. Sex determination mechanisms in reptiles are remarkably diverse, including well-characterised genetic and temperature-dependent sex determination. In rarer, but increasingly more commonly documented cases, sex can also be determined by a combination of the two, with temperature overriding the genetically determined sex. Thus, sex-by-temperature interactions is a mechanism that can be contextually labile, where reaction norms of sex against developmental environment might only be observable under certain conditions. We examine the effects of incubation temperature on hatchling sex in an oviparous lizard with clearly defined heteromorphic sex chromosomes presumed to determine sex solely on a genetic basis. We also test the repeatability of our results by replicating incubation experiments across 3 years. We show that warmer temperatures may override chromosomal sex and cause an overproduction of daughters. However, this effect was inconsistent among years, with high temperature only resulting in a daughter-significant bias in one year. Warm-incubated daughters were more efficient at converting yolk into tissue, which would allow for greater resource allocation to other fitness-related processes, such as growth. This suggests that thermolabile sex determination could be a trait under selection. More energy-efficient embryos also produced faster-growing offspring, suggesting that energy utilization patterns of the embryo were maintained into the juvenile stage, which could have important implications for the ontogenetic development and evolution of life histories.

## Teaser

**Summary:** Incubation temperature influences hatchling sex in a year-dependent manner in an oviparous reptile with heteromorphic sex chromosomes.

## INTRODUCTION

The determination of phenotypic sex is fundamentally important for development and life history of an individual with downstream consequences for sex ratio structure of populations (West et al., 2002). Unsurprisingly, much scientific effort has been allocated to understanding the diversity of ways in which sex is determined across the animal kingdom. Sex determination mechanisms of mammals and birds are highly conserved, where sex is determined genetically at conception by genes contained in sex chromosomes, with male heterogamety (XY) in mammals and female heterogamety (ZW) in birds. In contrast, reptiles show the full diversity of sex determination mechanisms (Janzen and Phillips, 2006; Bachtrog et al., 2014) and have proven vital to the study of vertebrate sex determination evolution (Shine, 1999; Shine et al., 2002; Warner and Shine, 2008b; Pen et al., 2010). In vertebrates, sex is generally determined either by genes at conception (genetic sex determination, GSD), or environmental cues (ESD), most commonly temperature during embryogenesis (temperature-dependent sex determination, TSD). Under some conditions, and in some taxa, the defining mechanism may also be a combination of genes and temperature, when environment can override genotypic sex (Shine et al., 2002; Valenzuela et al., 2003; Sarre et al., 2004; Hill et al., 2022). These types of systems have been hypothesised to be widespread in reptiles (Holleley et al., 2016), with documented cases becoming increasingly more common (see reviews Schwanz et al., 2020; Schwanz and Georges, 2021).

Frequency-dependent selection will typically act on a system where allocation towards one sex yields greater fitness returns, driven by sex ratio disequilibrium and differential reproductive costs associated with sons and daughters (Frank, 1990). In species with sex genes, sex is determined at conception and facultative alterations to mendelian parity are commonly caused by differential fertilization and/or mortality (Trivers and Willard, 1973; Krackow, 1995). In ESD species, the environment provides additional mechanisms for the adjustment of sex ratios, by parents adaptively altering nesting site and phenology, which can overproduce the sex giving the greatest return on investment as a function of sex-specific environmental optima (Doody et al., 2006; Warner and Shine, 2008a). Such sex-specific fitness differences are the most accepted evolutionary explanation of ESD (Charnov and Bull, 1977; Shine et al., 1995; Shine, 1999; Warner and Shine, 2008b). However, when directional environmental stimuli consistently maintain sex ratio bias in a population, frequency-dependent selection will favour a transition to GSD or random sex determination (Conover et al., 1992; Schwanz and Georges, 2021).

Assigning an adaptive value to a thermosensitive sex determination system can be difficult, especially in long-lived species where direct measurements of life-time fitness are impractical. We therefore often must accept early-life traits as predictors of survival and lifetime reproductive output. This makes predictors of growth rate prime candidates for fitness related traits, as it likely influences the time to, and size at, maturity (Angilletta, 2004). Such changes to growth rate could be caused by thermal effects on physiological efficiency by altering basal metabolic rate (Nord and Nilsson, 2011; Spencer and Janzen, 2014). This would allow a greater surplus of energy to be allocated to fitness-related processes, such as growth (Steyermark, 2002). If such a relationship between temperature and metabolic rate were to differentially affect male and female offspring, it would suggest that a thermosensitivity of sex determination is a trait under selection (Ligon et al., 2009).

Recently, environmental involvement in the sex determination of GSD species have received growing scientific attention and is proposed to be an ancestrally conserved trait in reptiles (Holleley et al., 2016; Schwanz and Georges, 2021). We test this hypothesis by examining the influence of incubation temperature on offspring sex in a lacertid lizard with clearly defined heteromorphic sex chromosomes, with female heterogamety (ZZ/ZW). We also duplicate the experiment across 3 years to determine whether the results are repeatable, or context-dependent. Our previous work on this system suggests the potential for labile sex allocation, where females overproduce daughters when mated to males of perceived higher quality (Olsson et al., 2005a,b). Furthermore, we examine whether the physiological efficiency of embryos is sex-specific by determining the conversion of yolk-to-tissue mass within each treatment, and if such effects are maintained post-hatching by influencing juvenile growth rates.

## RESULTS

During the 3-year study, a total of 81 clutches were produced including 574 fertilised eggs combined (with an average clutch size of 7.09 eggs) of which 561 hatched. Incubation duration was inversely related to incubation temperature (linear mixed model regression; *t*=–67.87, d.f.=501.1, *P*<0.0001), with eggs from 23°C hatching after an average of 50.6±0.2 days (40–56 days), 25°C eggs after 38.6±0.2 days (33–44 days), and 27°C eggs after 33.8±0.1 days (31–39 days). The eggs producing male or female hatchlings did not differ in size (linear mixed model regression; *t*=–0.693, d.f.=497.2, *P*=0.49), and egg mass was the strongest determinant of hatchling mass, regardless of incubation treatment (Tables 1 and 3). Male hatchlings from the 27°C were heavier compared their female counterparts when correcting for egg mass and egg mass-by-sex interaction, while an opposite trend, approaching significance, was found in the 23°C treatment (Table 3). However, the average weight differences between the sexes were miniscule (<0.5% difference in the 27°C treatment) and thus unlikely to be ecologically important.

**Table 1.**
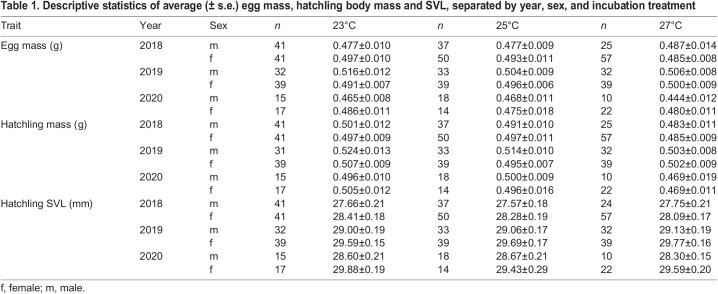
Descriptive statistics of average (± s.e.) egg mass, hatchling body mass and SVL, separated by year, sex, and incubation treatment

**Table 2.**
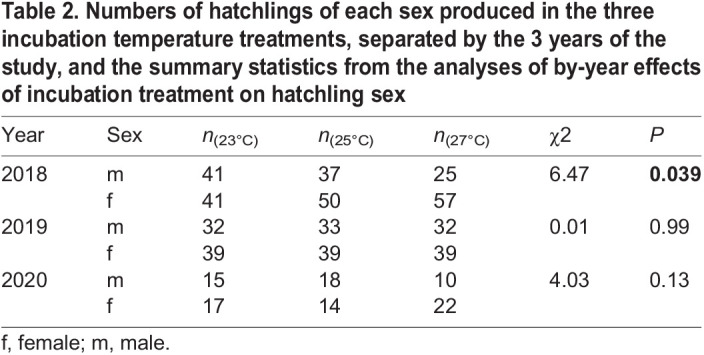
Numbers of hatchlings of each sex produced in the three incubation temperature treatments, separated by the 3 years of the study, and the summary statistics from the analyses of by-year effects of incubation treatment on hatchling sex

**Table 3.**
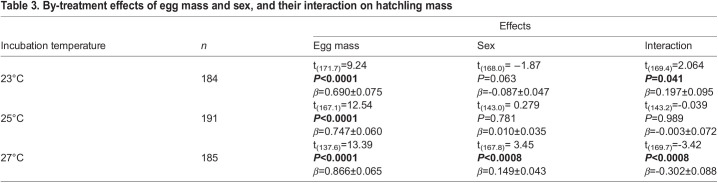
By-treatment effects of egg mass and sex, and their interaction on hatchling mass

**Fig. 1.**
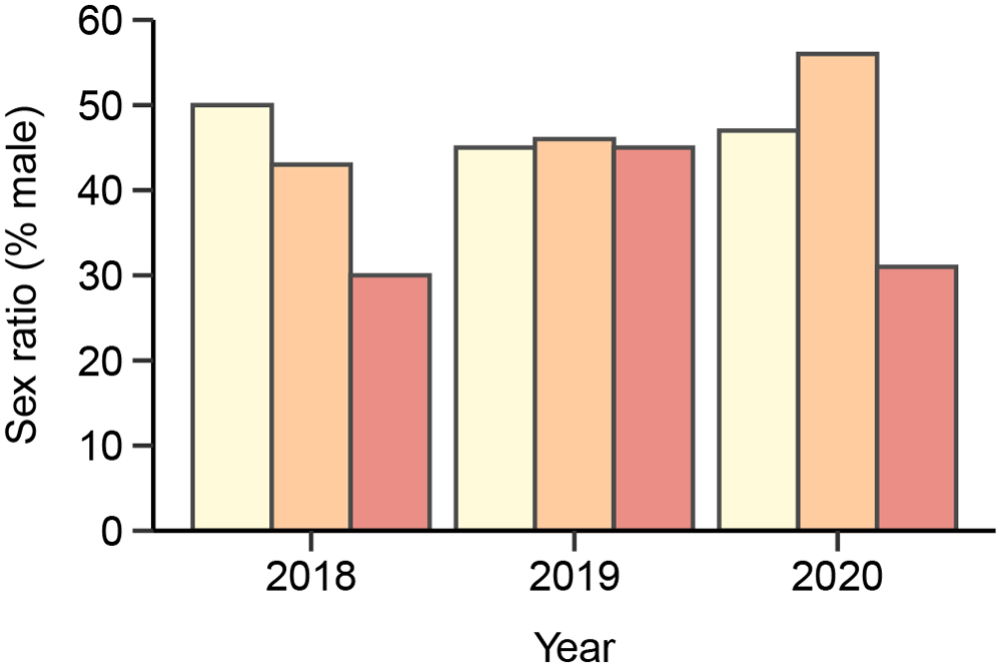
**Percent males produced by the three temperature treatments (23, 25, 27°C) in order of increasing colour saturation, and among the 3** **years of the study.**

Incubation temperature affected hatchling sex in a year-dependent manner. We observed a significant effect of incubation temperature on hatchling sex in 2018, when more female hatchlings were produced from eggs incubated at 27°C compared to those in 23°C, with an overall production of 70% and 50% females, respectively (Fig. 1, Table 2). Similar female bias, albeit non-significant, was observed in 2020, with a production of 69% females in 27°C against 53% females in 23°C. It is worth noting that the sample sizes in 2020 were about half of that in 2018 and 2019, which could have influenced the statistical power of the model and the results in this year. Sex ratios were unaffected by incubation treatments in 2019.

**Fig. 2.**
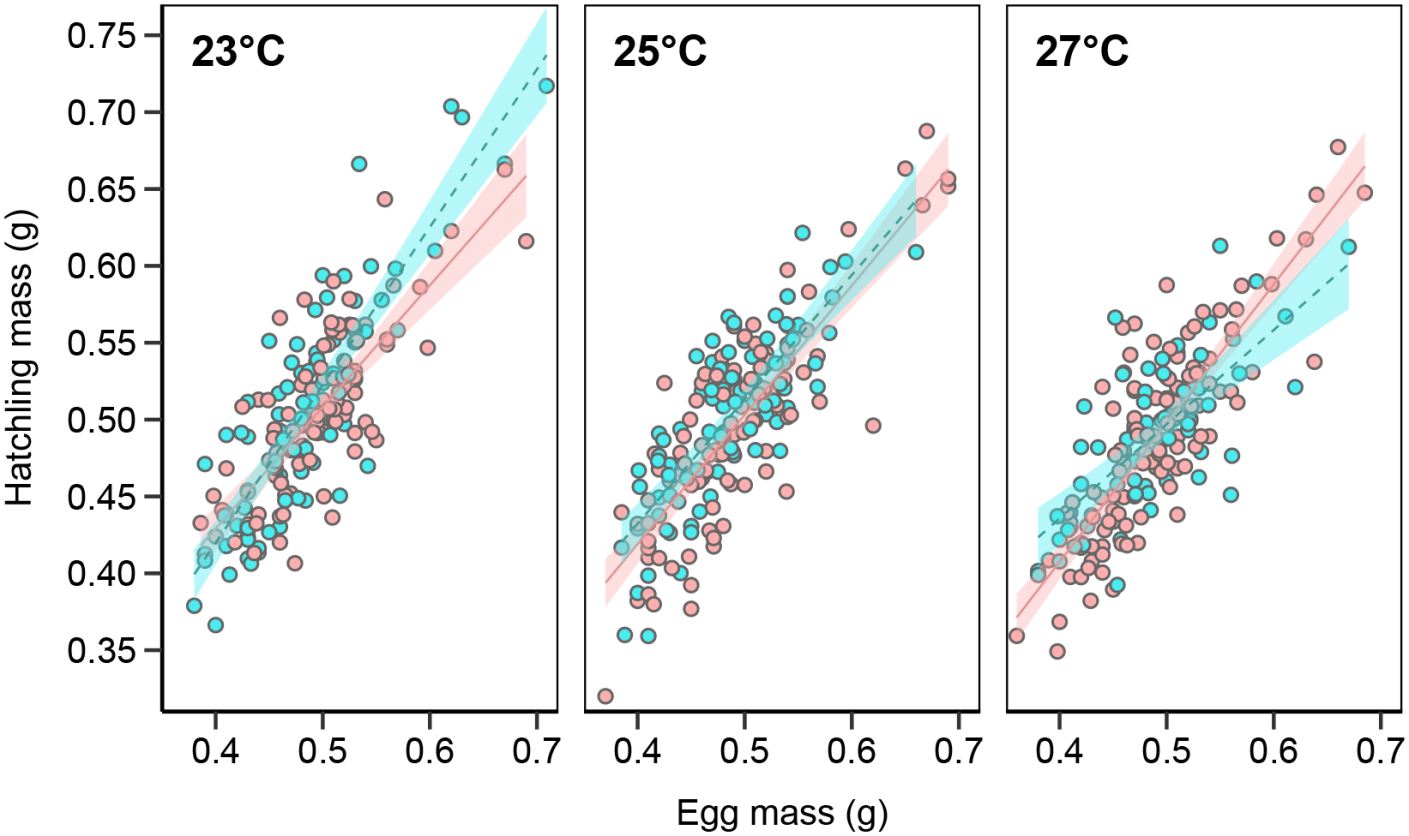
**Interaction plot of within-treatment relationships between egg mass and hatchling mass of male (blue) and female (red) hatchlings.** Slopes are based on linear regressions and shaded area denotes standard error range.

**Fig. 3.**
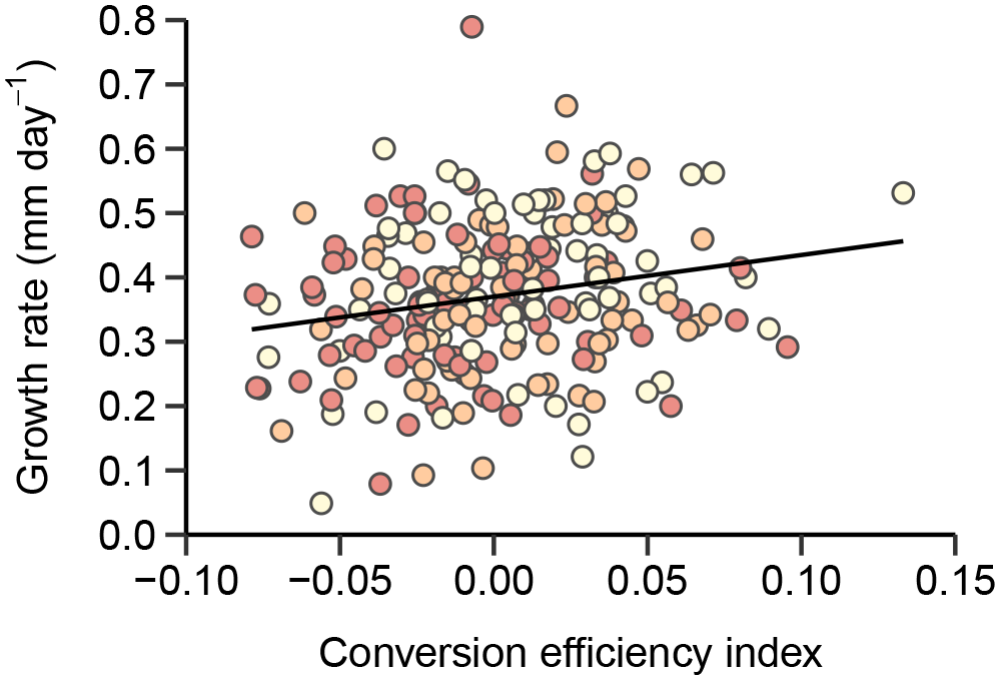
**Relationship between residual conversion efficiency of eggs and juvenile growth rate (mm/day).** Data points are separated by treatment (23, 25, 27°C) in order of increasing colour saturation.

Female hatchlings were significantly better than males at converting egg mass into hatchling mass when incubated at the temperature that overproduced female hatchlings (27°C), whereas the opposite was true for eggs incubated at 23°C, with males being the more efficient sex at converting egg mass into hatchling mass (Table 3, Fig. 2). This variation in energy efficiency extended beyond embryogenesis, with more energy-efficient eggs also producing faster growing juveniles (linear mixed model regression; *t*=2.23, d.f.=222.0, *P*=0.027; Fig. 3).

## DISCUSSION

We continue to uncover remarkable complexities of squamate sex determination. Recent research has highlighted the importance of this group for our understanding of sex determination mechanisms and their evolution (Shine et al., 2002; Quinn et al., 2007; Capel, 2017; Pennell et al., 2018; Schwanz and Georges, 2021). Our findings add to this research by providing the first robust evidence of thermolability in the sex determination of a lacertid lizard. Specifically, we show that high incubation temperature may override chromosomal sex causing an overproduction of daughters. However, this effect was inconsistent among years, with temperature only resulting in a significant sex-bias in one year of the study (and a trend in the same direction in another year). Such inconsistencies are not uncommon in sex-determination experiments in GSD species. Studies on birds are notable for showing context-dependent sex-ratio adjustments within and among studies, effects often varying between study year and population (Korsten et al., 2006; Henderson et al., 2014), potentially calling into question conclusions about the generality of sex ratio adjustments. The research of sex allocation in reptiles also show clear instances of a context-dependent reaction norm between environment and sex determination in GSD species. One such dependency has been observed as seasonal sex ratio variation within a population. The turtle with TSD, *Chrysemys picta*, experienced seasonal changes to the direction of sex ratio shifts where the same incubation temperature produced 72% males early in the season, and 76% females late in the season (Bowden et al., 2000). Such thermolability of sex determination has also been observed between altitudinally and latitudinally separated populations, as shown in both a viviparous and an oviparous lizard (Wapstra et al., 2004; Pen et al., 2010; Li et al., 2022). Thus, reaction norms of offspring sex against developmental temperature may themselves be labile, perhaps overlooked because replicated studies among contexts (in this case years) are rare. This can limit interpretations and predictions of reaction norms for sex-determination and sex-differentiation to the immediate conditions at which they are evaluated, conditions that might be predictive of the evolution of sex determination and its norm of reaction. This would be especially true in species that determine sex based on an interaction of genes and environment, where effects on offspring sex might only be observable as subtle biases under extreme environmental conditions.

In species with heteromorphic sex chromosomes where sex is determined at conception, biased sex ratios can be achieved by processes that cause differential fertilization by X and Y sperm (primary sex ratio bias), or by means of differential mortality of male and female embryos (secondary sex ratio bias). Because *Lacerta agilis* has clearly defined sex chromosomes with female heterogamety (Srikulnath et al., 2014; Lisachov et al., 2020), and we used a split-brood design with a very high hatching success, the overproduction of females cannot be explained by parental effects, differential fertilization, or sex-specific embryo mortality. A candidate proximate mechanism explaining the observed thermal involvement in the sex determination of *L. agilis* is an acquired thermosensitivity during sex differentiation causing a mismatch between genetic and phenotypic sex (sex reversal). The phenomenon of sex reversal has only been adequately demonstrated with a mismatch between phenotypic and genetic sex in three reptiles – all lizards – thus far, two oviparous [the bearded dragon, *Pogona vitticeps* (Quinn et al., 2007; Holleley et al., 2015; Castelli et al., 2020), the Eastern three-lined skink, *Bassiana duperreyi* (Shine et al., 2002; Radder et al., 2008; Quinn et al., 2009)], and one viviparous [the spotted snow skink, *Niveoscincus ocellatus* (Wapstra et al., 2004; Pen et al., 2010; Cunningham et al., 2017; Hill et al., 2018, 2022)]. Although there are only a few cases confirmed of sex reversal, phylogenetic inference has established TSD as an ancestral reptilian trait (Janzen and Phillips, 2006) and evidence of thermosensitive sex determining genes in GSD species to be ancestrally conserved (Valenzuela, 2007). This suggests that the capacity for sex reversals might be widespread in reptiles (see Holleley et al., 2016; Schwanz and Georges, 2021 for reviews). Recent discoveries of GSD and environmental effects (GSD+EE) from several species lend further weight to this hypothesis, including data from the Jacky dragon (*Amphibolurus muricatus*), the multi-ocellated racerunner (*Eremias multiocellata*), the common collared lizard (*Crotaphytus collaris*), the Japanese gecko (*Gekko japonicus*), and the yellow-bellied water skink (*Eulamprus heatwolei*) (Wang et al., 2015; Cornejo-Páramo et al., 2020; Wiggins et al., 2020; Whiteley et al., 2021; Li et al., 2022). Moreover, local variation in sex determination systems has recently been found in *P. vitticeps* and proposed to be the result of genetic adaptations to local environmental conditions (Castelli et al., 2020). The sand lizard population used in this study was experimentally founded on an island about 20 years ago. We have recently found evidence for increased plasticity in the reproductive biology of island-females compared to their mainland counterparts (Olsson et al., 2018). Future work should be dedicated to determining whether the observed sex bias is in fact due to sex reversal and whether a similar thermosensitive sex determination exist in mainland populations, or whether it is a locally acquired trait unique to this island population. If the latter is found to be true, this population will offer exciting opportunities to study the evolution of thermosensitive sex determination in reptiles.

The evolution of thermosensitivity in GSD species is likely to be under similar selective pressures as those in a TSD system. Sex genes may ensure balanced sex ratios under normal temperatures but can be overridden by extreme temperatures to overproduce the sex with greater potential fitness in the more extreme environment. One proposed mechanism of sex reversal is the acquisition of a thermosensitive gene dosage in the homogametic sex, in which extreme temperatures may inactivate the sex-determining gene (Quinn et al., 2007, 2011; Ezaz et al., 2009). In agreement with our results, all confirmed cases of reptile sex-reversal are unidirectional, the homogametic sex is the reversing one, producing XX males and ZZ females and causing a sex ratio bias towards the heterogametic sex. This is predicted by evolutionary theory where sex reversal is suboptimal or lethal in the heterogametic sex from second generation YY and WW chromosomal combinations (Bull, 1981; Schwanz et al., 2013).

Our split-brood design is arguably a robust approach for analysing among- and within-year thermal effects on sex and phenotype. Thus, the inconsistent thermosensitivity of sex determination in *L. agilis* can only be explained by some additional factor outside the scope of our experimental design. One candidate hypothesis is natural yearly temperature variation, with 2018 being a notable thermal outlier, and indeed the year in which we observed a significant female bias in the warmest incubation treatment. The average daily temperature in 2018 during the month of May – when females emerge from hibernation and most ovulations occur (Olsson and Madsen, 1996; Olsson et al., 2011a) – was over 5°C warmer compared to the two subsequent years. There is little experimental research of pre-ovipositional environmental effects on sex determination, but a sensitivity to sex hormone inhibitors has shown to be present at oviposition in some squamates and perhaps even earlier (Shine et al., 2007). Furthermore, maternal temperature prior to, and during egg production, has been observed in a skink to affect the thermolability of sex determination independent of incubation temperature (Schwanz, 2016). For such effects to explain our inconsistent results, pre-ovipositional influence must alter the thermosensitivity of sexual differentiation post-oviposition due to our split-brood design. The importance of hormonal or epigenetic effects should not be underestimated, especially in squamates where most embryos complete a substantial portion, about 25–40%, of their development prior to oviposition (Shine, 1983; DeMarco, 1993). This warrants future investigation of pre-ovipositional influence on downstream thermosensitivity of sex determination.

Regardless of what proximate cause(s) result in an overproduction of one sex, the selective pressure acting on GSD+EE species is likely similar to that in ESD species. Evolutionary explanations of ESD mainly rest upon the presence of sex-specific differential fitness linked to development conditions (Shine et al., 1995; Schwanz and Georges, 2021). We show that daughters incubated in the warmest, female-biased, treatment were more efficient at converting yolk into tissue mass. The opposite was true in the coolest incubation treatment, in which male embryos from large eggs were more efficient converters. If this is caused by a sex-specific differential energy efficiency, it would support the observed thermolability of sex determination as an adaptive trait. Energy efficiency, in this context, refers to the portion of the total energy budget utilised for basal metabolism and if reduced could allow for greater resource allocation to other fitness-related processes, such as growth (Steyermark, 2002; Ligon et al., 2009). Sex-specific responses to overwintering temperature, shown as differential metabolic expenditure and growth, have previously been observed in the painted turtle (*Chrysemys picta*), in which male and female hatchlings had different optimal overwintering temperatures (Spencer and Janzen, 2014). This was argued to be a proximate mechanism for the adaptive maintenance of TSD in this turtle by producing the sex best suited, metabolically, to the current environmental conditions. Similar effects have recently been shown in zebra finches (*Taeniopygia guttata*), in which incubation temperature differentially affect the basal metabolic rate of male and female hatchlings (Wada et al., 2015; Gurley et al., 2018). If such changes to basal metabolic rate were to extend beyond embryogenesis, it could have major implications for life history trade-offs throughout life, such as time to, and size at, maturity, and the capacity for future reproductive investments (Burton et al., 2011; Ben-Ezra and Burness, 2017). We observed exactly this; individuals produced from more energy-efficient embryos also grew at a faster rate after hatching, suggesting that the proposed effects on energy utilization could be extended into the juvenile – and perhaps later –  life stages. This would further support the hypothesis that the observed thermolabile sex determination in *L. agilis* is a trait under selection.

In conclusion, we provide the first evidence of a context-dependent thermolabile aspect to the sex determination of a lacertid lizard. We also show that this reaction norm might be maintained through selection by differential energy efficiency of male and female embryos. This hypothesis is further strengthened by our observations of more energy-efficient embryos producing faster-growing juveniles, suggesting prolonged benefits. Future studies will inevitably uncover the prevalence of reaction norms involved in sex determination of reptiles with sex genes. However, the increasing evidence of such reaction norms clearly highlight the necessity to consider these effects going forward. We also stress the importance of considering context when studying modes of sex determination, where snapshot studies might not be sufficient to draw meaningful conclusions of negative results, and the biological importance of positive results can easily be exaggerated.

## MATERIALS AND METHODS

### Study species, collection, and husbandry

The sand lizard (*L. agilis*) is a small (max 20 g) ground-dwelling oviparous lizard with one of the largest distributions of any reptile (Bischoff, 1984), reaching from Sweden in the north to Turkey in the south (Bülbül et al., 2019), and stretching from western France to Mongolia. This makes *L. agilis* the most northern-occurring oviparous lizard in Europe. Our study population was experimentally founded about 20 years ago on a small island (St. Keholmen) located on the southwestern coast of Sweden (57°29′ N 11°56′ E) (see Lindsay et al., 2020), which is near the northern limit of the species' range. Females produce a single clutch of 4–15 eggs each year, but can, when exposed to optimal conditions, produce a second clutch (Olsson and Shine, 1997a,b). We collected adult female sand lizards by noose or by hand after Spring emergence in early May of 2018–2020 and palpated for eggs. Egg-carrying females were brought back to the laboratory and housed in cages (500×400×350 mm) with a flat basking rock on a moistened sand substrate to act as a favourable oviposition site. The ambient temperature was set to fluctuate daily between 15 and 20°C, simulating natural conditions, and a 40W spotlight positioned over the basking rock allowed behavioural thermoregulation to 40°C body temperature. Females were fed live meal worms dusted with calcium and multivitamins and provided with water *ad libitum*.

### Egg collection and incubation

Gravid females (*n*_(2018)_=33, *n*_(2019)_=29, *n*_(2020)_=16) were closely monitored and eggs were removed from the cage within hours of oviposition. In the 3 years combined, females laid a total of 574 fertilised eggs (*n*_(2018)_=258, *n*_(2019)_=218, *n*_(2020)_=98). Three females produced a second clutch in 2019, while no second clutches were produced in the two other years. Due to the nature of this longitudinally studied population, some females were included in more than 1 year, with a total of ten females included in more than 1 year (nine in 2018, ten in 2019, two in 2020). At oviposition, eggs were removed from the female cage and brushed clean from sand and moisture, weighed to the nearest 0.01 g and placed individually in plastic cups half-filled with moist vermiculite (1:8 water to vermiculite by volume). The cups were sealed with plastic cling wrap and rubber bands to prevent moisture loss. To test the influence of incubation temperature on the developing embryo, we divided sibling eggs among three constant incubation temperature treatments in accordance with a split-brood design which minimises the risk of confounding parental and treatment effects (Via, 1993). The temperatures used were 23, 25 and 27°C, based around the optimal incubation temperature of *L. agilis*; 25°C that minimise developmental abnormalities and asymmetries (Zakharov, 1989). Although natural nests are unlikely to experience such warm conditions consistently throughout incubation, this species practice uterine retention in cool years (Shine et al., 2017; Olsson et al., 2018). Furthermore, studies on other oviparous lacertids show that the average time the embryo spends *in utero* has been estimated to be about half of total time of embryogenesis (Shine, 1983). Such retention would result in the embryo experiencing longer periods of high maternal temperatures associated with behavioural thermoregulation that easily can exceed the incubation temperatures used in this study (Bauwens et al., 1995). Incubating eggs were rotated among three shelves in each incubator weekly to minimise the effect of thermal gradients inside the incubator and were monitored daily for pipping (first sign of eggshell rupture).

### Hatchling morphological measurements

A total of 561 eggs hatched (*n*_(2018)_=251, *n*_(2019)_=214, *n*_(2020)_=96), with a hatching success between 97–98% within and among years. On the day of hatching, hatchlings were blotted dry, brushed clean of vermiculite and weighed (±0.01 g). SVL was measured using a ruler (±1 mm). Hatchling sex was determined within hours of hatching by gently pressing on both sides of the tail base using modified forceps with tips bend to a V-shape to observe the presence or absence of hemipenes (Harlow, 1996; Olsson and Shine, 2003). This sexing method has 100% repeatability in this species (Olsson et al., 2004; 2005a, 2011b). The length of incubation was defined as the time between oviposition and hatching.

### Post-hatching husbandry and growth

In the last 2 years of the study, we examined the effects of embryonic development and environment on subsequent juvenile growth patterns. Hatchlings were placed in cages of the same size and design as the adults, but with more rocks to allow space for thermoregulation. Initially, a maximum of ten juveniles were housed per cage, subsequently lowered to five after a growth period of about 2 weeks. Juveniles were fed daily with small crickets (2–6 mm) and mealworms, all dusted with vitamins and minerals as for adults. The amount of food was always in excess, allowing juveniles to feed *ad libitum*. Water was also provided *ad libitum*, with cages additionally misted with water daily. We remeasured juveniles for SVL that had grown for a minimum of 20 days, which excluded seven individuals that had not reached this threshold at the end of the experiment. This lower threshold was set to minimise the influence of the differential growth and feeding ecology during the first days after hatching when residual yolk is metabolised as a source of energy (Troyer, 1987). Juveniles included in analyses were remeasured after between 20–63 days, with a total of 230 juveniles measured (*n*_(2019)_=165, *n*_(2020)_=65). The upper limit of growth duration was the logical end of the experiment when juveniles were returned to the wild given ample time to prepare for hibernation. Growth during this life stage is generally linear in reptiles (Andrews, 1982; Li et al., 2013), and thus using growth rate (see below) as a measure of growth is used to account for the variation in growth duration.

### Data analysis

All statistical models were fitted in R v4.1.2 (R Core Team, 2021). Generalised linear mixed models (GLMM) were fitted using the *glmer* function from the *lme4* package (Bates et al., 2014), where test statistics were estimated for significance at alfa=0.05 by likelihood ratio tests. Linear mixed models (LMM) were fitted using the *lmer* function from the *lme4* package with denominator degrees of freedom, *t*-statistics and *P*-values for the LMMs derived from Kenward-Roger approximations using the *lmerTest* package (Kuznetsova et al., 2017). Maternal identity was included as a random effect in all mixed models to account for the pseudo-replication of siblings.

To test the influence of incubation temperature on hatchling sex, we fitted a GLMM with binomial error distributions with sex as response and incubation temperature as fixed factor, with maternal identity as random effect. Pooling all years and examining the effects of year and year-by-treatment did not reveal any significant influence on sex. One of the years of the study (2018) was an extreme thermal outlier, and we therefore decided to analyse years separately to avoid spurious interactions with other predictors, including incubation temperature.

To test whether thermal effects on hatchling sex might be sex-specific and potentially an adaptive trait, we examined sex-effects on yolk-to-tissue conversion from eggs to hatchlings. Because sand lizard eggs incubated at the same temperature can differ in incubation duration (Olsson et al., 2018), and lizards in general, including most lacertids, commonly show nonlinear developmental responses to constant incubation temperatures (Li et al., 2013), we analysed each treatment separately. We did this by fitting LMMs with hatchling mass as response and egg mass and sex, and their interaction, as fixed factors with maternal identity and year as random factors. Such sex-specific conversion efficiency is likely to cause short-term effects, like earlier hatching by completing development faster, or result in a larger body size at hatching from less thermally induced energy waste. However, long-term effects of conversion efficiency would likely have greater impact on individual fitness, and we also therefore examined whether more energy efficient embryos produced faster growing juveniles, which could have major implications on life history ontogeny and long-term evolution. Growth rate was calculated as the total SVL growth (mm) divided by the number of days of growth. Conversion efficiency was estimated from residuals from a regression of hatchling mass on egg mass. An LMM with growth rate as response, conversion efficiency as fixed effect and incubation temperature as covariate was modelled, with maternal identity and year as random factors.
